# Spinal NKCC1 blockade inhibits TRPV1-dependent referred allodynia

**DOI:** 10.1186/1744-8069-3-17

**Published:** 2007-06-30

**Authors:** Mark H Pitcher, Theodore J Price, Jose M Entrena, Fernando Cervero

**Affiliations:** 1 McGill University, Anesthesia Research Unit, Faculty of Medicine, Faculty of Dentistry and McGill Centre for Research on Pain, 3655 Sir William Osler, McIntyre Medical Bldg 1207, Montreal, QC, H3G 1Y6, Canada; 2 University of Granada Faculty of Medicine, Department of Pharmacology and Neurosciences Institute, Avenida de Madrid 12, E-18012, Granada, Spain

## Abstract

**Background:**

The Na^+^, K^+^, 2Cl^- ^type I cotransporter (NKCC1) and TRPV1 receptors, at the level of the dorsal horn, have been implicated in mediating allodynia in response to an inflammatory insult. The NKCC1 cotransporter regulates intracellular [Cl^-^] and thus the magnitude and polarity of GABA_A _receptor responses in neurons. TRPV1 receptors transduce diverse chemical and natural stimuli in nociceptors and are critical for inflammatory hyperalgesia.

**Results:**

Here we have tested the role of spinal NKCC1 cotransporters and TRPV1 receptors in referred allodynia in a model of visceral hyperalgesia in mice. Intrathecal (IT) injection of the NKCC1 inhibitor bumetanide (BUM, 1 nmol) inhibited referred, abdominal allodynia evoked by an intracolonic capsaicin injection. BUM was effective when injected IT either before or up to 4 hrs after the establishment of referred allodynia. The TRPV1 antagonist AMG 9810 (1 nmol) also inhibited referred allodynia in this model suggesting the involvement of an endogenous TRPV1 agonist in the dorsal horn in referred allodynia. In support of this suggestion, the endovanilloid TRPV1 agonist, narachidonoyl- dopamine (NADA, 1 or 10 nmol, IT) evoked stroking allodynia in the hindpaw that was blocked by co-treatment with AMG 9810 (1 nmol). The TRPV1-dependent stroking allodynia caused by NADA appeared to be functionally linked to NKCC1 because BUM (1 nmol) also inhibited NADA-evoked stroking allodynia.

**Conclusion:**

Our findings indicate that spinal NKCC1 and TRPV1 are critical for referred allodynia mediated by a painful visceral stimulus. Moreover, they suggest that endogenous TRPV1 agonists, released in the CNS in painful conditions, might stimulate TRPV1 receptors on primary afferents that, in turn, play a role in increasing NKCC1 activity leading to allodynia.

## Background

Intracellular chloride concentration in neurons is maintained by members of the Na^+^, K^+^, 2Cl^- ^(NKCC) and K^+^, Cl^- ^(KCC) families of cation-chloride cotransporters [1]. The NKCC proteins accumulate chloride intracellularly and, in dorsal root ganglion (DRG) neurons, it is the primary mechanism that sets the reversal potential for chloride conductance through GABA_A_-receptors (GABA_A_R) [2,3]. Unlike most CNS neurons, DRG neurons maintain depolarizing responses to GABA_A_R agonists throughout postnatal development [2,3]. These depolarizing GABA_A_R responses are dependent on NKCC1 expression because depolarizing GABA_A_R responses in DRG neurons are reduced in NKCC1^-/- ^mice [3]. It has been suggested that some pain states might involve enhancements of primary afferent GABA_A_R responses such that the normal small GABAergic epolarization of these fibers is augmented to the point that it induces a direct activation of spinal nociceptors [4-7]. This has led to the proposal that NKCC1 is responsible for the increase in intracellular chloride that could mediate GABA_A_R-dependent depolarization above threshold for spike generation in nociceptors [5-7]. In support of this hypothesis, it has been shown that NKCC1^-/- ^mice display reduced responses to noxious heat as well as reduced touch-evoked pain [3,8]. Furthermore, intrathecal delivery of the NKCC1 blocker bumetanide (BUM) inhibits nocifensive behavior in phase II of the formalin test [9] and mechanical allodynia induced by capsaicin injection into the hindpaw [10] in rats. Finally, intracolonic capsaicin injection stimulates a rapid and transient increase in spinal phosphorylated NKCC1 and a long lasting increase in trafficking of NKCC1 protein to the plasma membrane [11]. Taken together these findings indicate that NKCC1 might play an important role in inflammatory and tissue damage pain.

In naive animals, Aα-fiber stimulation causes a GABA_A_R-dependent primary afferent depolarization (PAD) of nociceptors leading to a decrease in pain transmission in the spinal dorsal horn [7,12]. In inflammatory conditions Aβ-fibers are capable of directly exciting nociceptors via a GABAergic mechanism causing antidromic (termed dorsal root reflexes, DRRs) and orthodromic firing of nociceptors [13-16]. This process has been proposed as a mechanism of inflammation- or injury-evoked allodynia. Because it is dependent on depolarizing GABA_A_R responses, NKCC1 is a logical molecular candidate for mediating this effect [5-7]. Here we have tested the hypothesis that spinal NKCC1 mediates referred allodynia in response to a visceral inflammatory stimulus. TRPV1 receptors in the CNS, likely localized on primary afferent terminals in the dorsal horn, have recently been identified as an important target for inflammatory allodynia [17]. Hence, we have also tested the hypothesis that spinal TRPV1 receptors are involved in referred allodynia and we have investigated a possible link between spinal TRPV1-dependent allodynia and NKCC1. Our findings demonstrate that spinally applied inhibitors of NKCC1 and TRPV1 attenuate referred allodynia evoked by a painful visceral stimulus and show that spinally applied TRPV1 agonists cause allodynia that is likewise inhibited by NKCC1 blockade.

## Results

### Spinal NKCC1 blockade inhibits intracolonic capsaicin-evoked referred, abdominal allodynia and hyperalgesia

**Figure 1:**
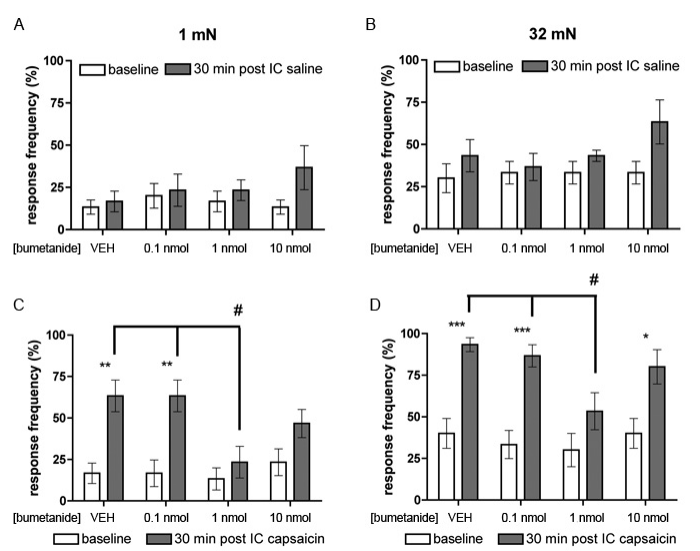
**NKCC1 inhibitor bumetanide blocks intracolonic capsaicin-evoked referred allodynia and hyperalgesia**. Three doses of bumetanide or vehicle (VEH) were injected intrathecally (IT) and saline or capsaicin was injected into the colon 5 min later. A and B) Baseline response frequencies to abdominal 1 mN and 32 mN von Frey hair application are shown as well as 30 min after the intracolonic (IC) saline injection. C and D) Response frequencies to abdominal 1 mN (C) and 32 mN (D) von Frey hair application 30 min after the IC capsaicin injection are shown in comparison to baseline von Frey response frequencies. Stars (*) indicate significant effects vs. baseline (two-way anova); number signs (#) indicate significant effects of IT bumetanide injections (one-way anova, # or * p < 0.05, ** p < 0.01, *** p < 0.001; n = 6 per group).

Work from this laboratory has shown previously that an intracolonic capsaicin injection causes a transient increase in NKCC1 phosphorylation and a sustained increase in plasma membrane localization of NKCC1 in the spinal dorsal horn [11]. Here we have tested the hypothesis that NKCC1 is functionally linked to referred allodynia and hyperalgesia in this model using spinal application of the NKCC1 inhibitor bumetanide (BUM). We first sought to determine the effects of increasing doses of intrathecal (IT) BUM by itself and the effects of IT BUM on referred (abdominal) allodynia and hyperalgesia 0.5 hrs following an intracolonic capsaicin (0.1%) injection. In this model referred allodynia peaks at 20 min post capsaicin injection, is sustained at this peak level for 6 hrs, and referred allodynia is evident for at least 24 hrs [18,19]. BUM at 0.1, 1 and 10 nmol had no effect on response frequencies to 1 (Fig 1A) or 32 mN (Fig 1B) stimulation of the abdomen. When BUM, at the same doses, was injected IT 5 min prior to intracolonic capsaicin it dose-dependently inhibited referred allodynia (1 mN stimulation of the abdomen, Fig 1C) and 1 nmol BUM inhibited referred hyperalgesia (32 mN stimulation of the abdomen, Fig 1D). BUM (1 nmol) had no effect on nocifensive responses evoked by intracolonic capsaicin (VEH = 88.0 ± 10.7 s, BUM = 78.5 ± 13.6 s) nor did it influence the latency to first nocifensive behavior (VEH = 77.7 ± 17.4 s, BUM = 57.8 ± 12.9 s).

**Figure 2:**
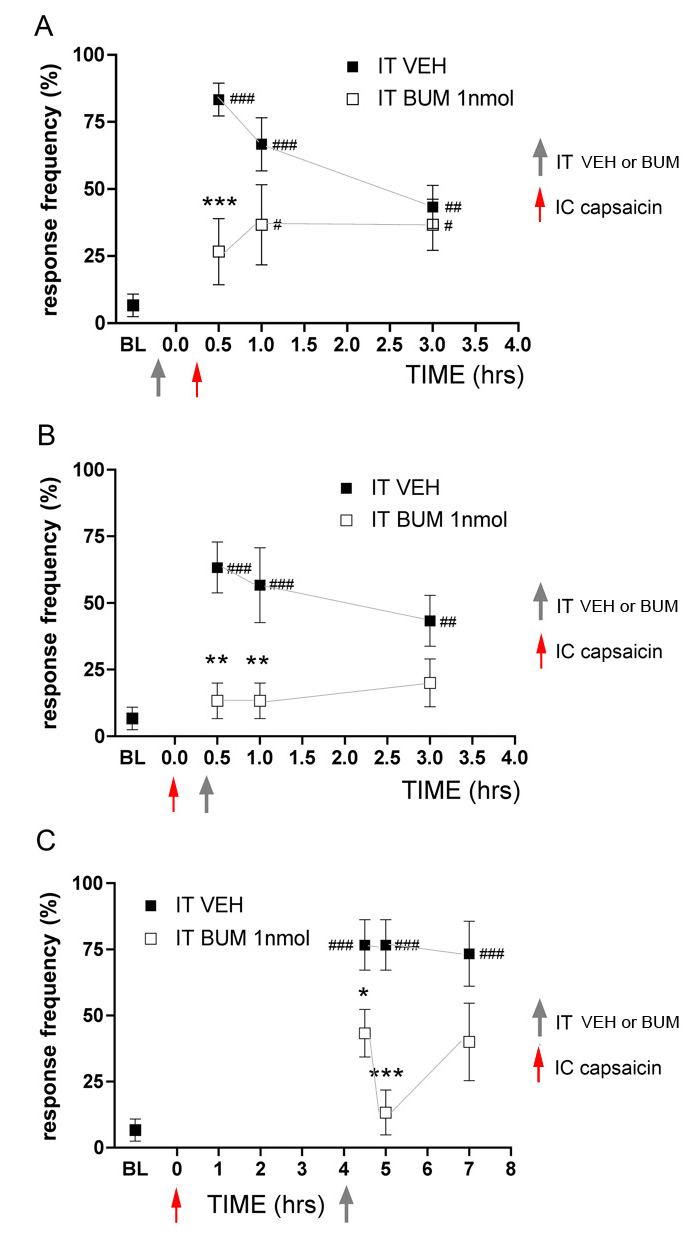
**Time course of NKCC1 inhibitor bumetanide blockade of intracolonic capsaicin-evoked referred allodynia**. Bumetanide (BUM) or vehicle (VEH) was injected intrathecally (IT) 5 min prior to intracolonic (IC) capsaicin (A) or 20 min (B) or 4 hrs (C) after IC capsaicin. Response frequencies to 1 mN von Frey hair application to the abdomen are shown at the indicated time points post IC capsaicin. Stars (*) indicate significant effects of BUM vs. VEH (two-way anova) while number signs (#) indicate significant effects vs. baseline (BL) at a given time point (one-way anova, # or * p < 0.05, ## or ** p < 0.01, ### or *** p < 0.001; n = 6 per group).

Next we addressed the time course of IT BUM effects on the inhibition of intracolonic capsaicin-evoked referred allodynia. BUM (1 nmol) was injected IT 5 min prior to intracolonic capsaicin application and significantly inhibited responses to 1 mN abdominal stimulation at 0.5 hrs (Fig 2A). However, BUM injected mice developed referred allodynia by 1 hr and this was maintained at 3 hrs illustrating that, while spinal NKCC1 blockade inhibits referred allodynia, it does not prevent its subsequent development (Fig 2A). If spinal NKCC1 activity contributes to the maintenance of referred allodynia we would predict that IT BUM injection following intracolonic capsaicin would inhibit abdominal allodynia. When BUM (1 nmol) was injected IT 20 min following intracolonic capsaicin it inhibited referred allodynia 0.5 and 1 hr post capsaicin (Fig 2B). Three hrs following intracolonic capsaicin the BUM group was not different from baseline (did not display referred allodynia) but was also not different from mice that received IT VEH. When BUM (1 nmol) was injected IT 4 hrs post capsaicin it inhibited referred allodynia 0.5 and 1 hr post BUM injection (Fig 2C). At the 7 hr time point the BUM group was not statistically different from baseline (did not display referred allodynia) but was also not different from the IT VEH group. These findings indicate that spinal NKCC1 blockade either before or after intracolonic capsaicin is capable of inhibiting referred allodynia.

### Spinal TRPV1 receptors regulate referred allodynia: role of NKCC1

**Figure 3:**
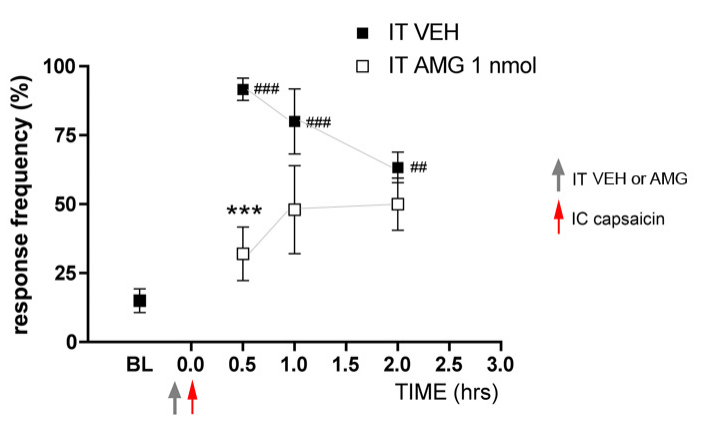
**Spinal TRPV1 antagonism inhibits intracolonic capsaicin-evoked referred allodynia**. The TRPV1 antagonist AMG 9810 (AMG, 1nmol) was injected intrathecally (IT) 5 min prior to intracolonic (IC) capsaicin. Response frequencies to abdominal von Frey hair (1 mN) stimulation at the indicated time points post intracolonic capsaicin are shown. Stars (*) indicate significant effects of AMG vs. VEH (two-way anova) while number signs (#) indicate significant effects vs. baseline (BL, one-way anova) at a given time point (## p < 0.01, ### or *** p < 0.001; n = 6 per group).

The role of TRPV1 in inflammatory hyperalgesia is well established and appears to involve a peripheral mechanism in which TRPV1 receptors are sensitized and respond to lower temperature stimulation than their basal thresholds [20,21]. It is also known that TRPV1 is involved in inflammatory mechanical hyperalgesia and, interestingly, recent studies have suggested that this effect is due to TRPV1 receptors in the CNS, likely on primary afferents in the dorsal horn [17]. Hence, we hypothesized that spinal TRPV1 receptors might be involved in referred allodynia in response to intracolonic capsaicin. The TRPV1 antagonist AMG 9810 (1 nmol) [22] was injected IT 5 min prior to intracolonic capsaicin and referred allodynia (1 mN stimulation of the abdomen) was measured at 0.5, 1.0 and 2.0 hrs. Mice that received an IT AMG 9810 injection did not differ from baseline at 0.5, 1.0 or 2.0 hrs post intracolonic capsaicin injection while mice that received an IT VEH injection displayed referred allodynia at all time points (Fig 3). The AMG 9810 group was significantly decreased vs. VEH injected animals at the 0.5 hr time point but not at 1.0 and 2.0 hrs (Fig 3). This observation illustrates that spinal TRPV1 receptors are involved in referred allodynia evoked by an intracolonic capsaicin injection.

**Figure 4:**
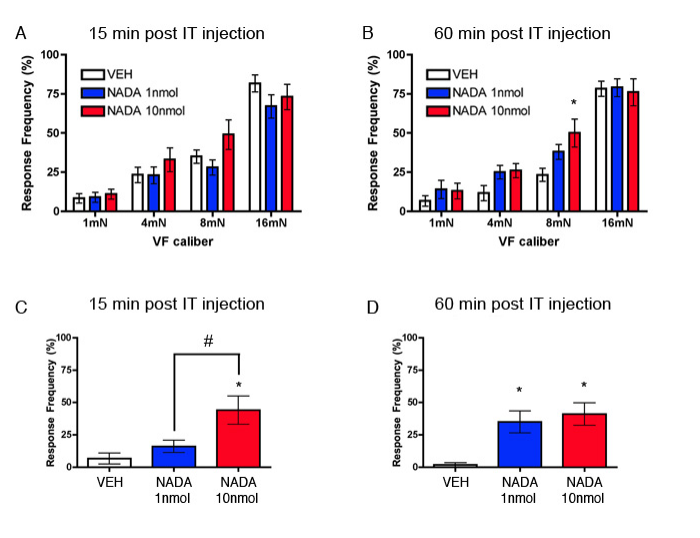
**Spinal TRPV1 agonist causes stroking allodynia in the hindpaw**. The endogenous TRPV1 agonist NADA (1 or 10 nmol) or vehicle (VEH) was injected intrathecally (IT) and the hindpaws were stimulated with von Frey hairs of the indicated calibers 15 (A) and 60 min (B) or a cotton bud 15 (C) and 60 min (D) after the IT injection. Stars (*) above columns indicate significant effects of NADA vs. VEH while number signs (#) above horizontal bars indicate significant effects of NADA 1 nmol vs. NADA 10 nmol (# or * p < 0.05; n = 10 per group). Panels A and B, two-way anova; panels C and D one-way anova.

**Figure 5:**
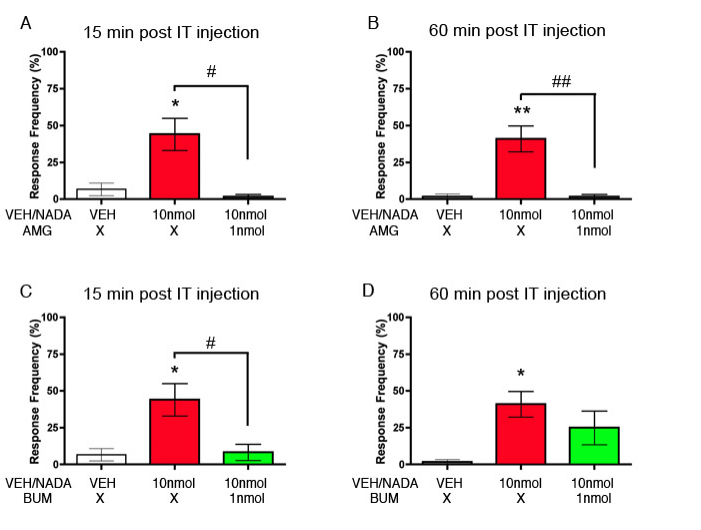
**NADA-evoked stroking allodynia is TRPV1-dependent and inhibited by bumetanide**. The endogenous TRPV1 agonist NADA (10 nmol) or vehicle (VEH) was injected intrathecally (IT) with or without a co-injection of the TRPV1 antagonist AMG 9810 (1 nmol) and the hindpaws were stimulated with a cotton bud 15 (A) and 60 min (B) after the IT injection. NADA (10 nmol) or VEH was injected IT with or without a co-injection of the NKCC1 antagonist bumetanide (BUM, 1 nmol) and the hindpaws were stimulated with a cotton bud 15 (C) and 60 min (D) after the IT injection. Response frequencies to the cotton bud stimulation are shown. Stars (*) above columns indicate significant effects of NADA vs. VEH while number signs (#) above horizontal bars indicate significant effects of antagonists (one-way anova, * or # p < 0.05, ** or ## p < 0.01; n = 6 per group).

The previous findings cited above and our present observation with AMG 9810 suggests that an endogenous TRPV1 agonist is active in the spinal dorsal horn and is involved in regulating mechanical nociceptive thresholds. One such candidate endogenous TRPV1 agonist is n-arachidonoyl-dopamine (NADA) [23]. NADA is a potent TRPV1 agonist, causes hyperalgesia in vivo [23,24] and exists in pharmacologically relevant concentrations in the CNS [23,25]. We hypothesized that IT NADA would stimulate allodynia and/or hyperalgesia in the hindpaw. 1 or 10 nmol NADA or VEH was injected IT and the hindpaw was stimulated with 1 – 16 mN von Frey hairs 15 and 60 min following the IT injection. 1 nmol NADA had no effect on von Frey response frequency at any time point and 10 nmol NADA evoked hyperalgesia to an 8 mN stimulus only at the 60 min time point (Fig 4A and 4B). We also measured stroking allodynia as measured by stroking the hindpaw with a cotton bud. Mice injected with 10 nmol NADA displayed stroking allodynia at both 15 and 60 min following IT NADA (Fig 4C and 4D). Mice injected with 1 nmol NADA had stroking allodynia only at the 60 min time point (Fig 4D). We did not observe nocifensive behaviors in response to NADA at either dose (data not shown). This finding establishes that the TRPV1 agonist NADA evokes stroking allodynia in the hindpaw following spinal application. We next tested the ability of the TRPV1 antagonist AMG 9810 (1 nmol) to block the effect of NADA (10 nmol). AMG 9810 completely blocked NADA-evoked stroking allodynia at 15 and 60 min post IT injection (Fig 5A and 5B) indicating that the effects of NADA are TRPV1-mediated.

Finally, we hypothesized that NADA-evoked stroking allodynia would be attenuated by the NKCC1 blocker BUM. When BUM (1 nmol) was co-injected with NADA stroking allodynia was completely blocked 15 min following IT injection (Fig 5C). Sixty min post injection BUM injected mice did not differ from VEH but response frequency to a stroking stimulus also did not differ from NADA injected animals (Fig 5D). Hence, the NKCC1 inhibitor BUM reduces spinally mediated TRPV1-dependent stroking allodynia.

## Discussion

In naive animals Aβ-fiber stimulation inhibits C-fiber activity via a mechanism called primary afferent depolarization (PAD) [7,12]. PAD is mediated by GABA_A_Rs on primary afferents where GABA has a depolarizing action. The molecular mechanism for depolarizing GABA_A_R currents appears to be expression of NKCC1 in DRG neurons leading to a high intracellular Cl^- ^concentration and Cl^- ^efflux through the GABA_A_R channel upon pharmacological stimulation [2,3]. We and others have proposed that GABAergic mechanisms may explain allodynia in inflamed conditions such that NKCC1 activity increases leading to a large depolarization in C-fibers, causing spiking, in response to Aβ-fiber stimulation [5-7]. Several lines of evidence support this claim: 1) following an inflammatory stimulus, lamina I neurons acquire a novel Aβ-fiber input that is GABAergic in nature [13], 2) stroking allodynia following capsaicin injection into the hindpaw is absent in NKCC1 knockout mice [8], 3) spinal application of the NKCC1 blocker BUM inhibits intraplantar capsaicin-evoked, secondary mechanical allodynia in rats [10] and 4) a painful visceral stimulus increases dorsal horn NKCC1 phosphorylation and membrane trafficking [11], both of which are mechanisms known to increase NKCC1 activity and intracellular Cl^- ^concentrations [1,26]. BUM also inhibits formalin  induced nocifensive behaviors in the second phase of this model through both a spinal and peripheral mechanism [9] although the relevance of these findings to allodynia are not clear. Here we have added to this line of evidence by demonstrating that spinal application of BUM inhibits referred allodynia evoked by capsaicin injection into the colon. Taken together, these observations indicate that NKCC1 is an important regulator of touch-evoked pain in inflammatory conditions and that small molecules that inhibit this cotransporter might have clinical utility for allodynia in humans.

Some pharmacological considerations deserve attention with respect to IT BUM. The anti-hyperalgesic (32 mN abdominal stimulation) effect of BUM was limited to a narrow dose range (1 nmol). This is expected because BUM possesses only roughly a 10–100 fold selectivity for NKCC1 vs. KCC2 [27]. Inhibition of KCC2 by BUM would be expected to have the opposite effect on touch-evoked responses because decreased KCC2 expression (analogous to pharmacological inhibition) or KCC2 blockade with DIOA reverses the polarity of GABA_A_R responses in a subset of lamina I and II neurons, leading to allodynia which has been proposed as a mechanism of neuropathic pain [28]. KCC2 is not expressed by DRG neurons [29] so this consideration applies only to intrinsic dorsal horn neurons. We focused on the 1 nmol dose in the rest of the experiments because of the KCC2 consideration stated above. While pretreatment with BUM inhibited referred allodynia it did not block its development. This finding likely reflects the long-lasting nociceptive stimulus evoked by intracolonic capsaicin [19] and illustrates that inhibition of NKCC1 is not sufficient to block the development of referred allodynia. On the other hand, BUM did effectively inhibit referred allodynia after its establishment both at 20 min and 4 hrs post intracolonic capsaicin injection suggesting that NKCC1 inhibitors might be clinically useful in cases of established inflammatory allodynia.

The TRPV1 receptor is expressed by a subset of DRG neurons that project to lamina I and II of the dorsal horn [30]. The majority of these neurons also express NKCC1 [29], hence, we reasoned that spinal TRPV1 receptors might be involved in regulating NKCC1 activity. The role of TRPV1 receptors in thermal hyperalgesia is well established [20,21] and, recently, it has become clear that spinal TRPV1 receptors play a role in allodynia [17]. Our findings show that spinal application of AMG 9810, a potent and highly selective TRPV1 antagonist, inhibits referred allodynia in response to intracolonic capsaicin. This suggests that an endogenous TRPV1 agonist is active in this model to evoke or maintain allodynia. One such potential agonist is the endogenous vanilloid/cannabinoid NADA. NADA is found in pharmacologically relevant concentrations throughout the CNS [23] and its synthesis is regulated by neural activity [25]. NADA causes nocifensive behaviors and hyperalgesia when it is applied in the periphery [23,24] but its behavioral effect when injected into the spinal cord has not been explored. IT NADA did not cause nocifensive behaviors and had only a minor effect on punctate hyperalgesia (at 8 mN), however, it caused a robust stroking allodynia that was TRPV1-dependent. To our knowledge this is the first demonstration that an endogenous TRPV1 agonist causes allodynia when applied directly to the spinal cord further supporting the hypothesis that CNS TRPV1 receptors are critical for inflammatory allodynia [17].

NADA-mediated stroking allodynia was inhibited by the NKCC1 inhibitor, BUM, suggesting a role for NKCC1 in this effect. In NKCC1 knockout mice, hindpaw inflammation-mediated punctate hyperalgesia (von Frey) is preserved but stroking allodynia (cotton bud) is absent [8]. Our current observations with NADA and BUM are consistent with these findings from NKCC1 knockout mice insofar as stroking (or dynamic) allodynia appears to be the modality most affected in the hindpaw. It is unclear why punctate hyperalgesia and/or allodynia are not affected, however, dynamic allodynia has been suggested to be reflective of an interaction between Aβ- and C-fibers in the CNS following a sensitizing stimulus in experimental pain or in clinical pain in humans [31,32]. Mechanisms for increasing the activity of NKCC1 in primary afferent terminals have not been identified. In other CNS areas stimuli such as mGluR agonists and calcium-sensitive kinases are known to increase NKCC1 phosphorylation and activity [33]. TRPV1 activation stimulates extracellular regulated kinase 1&2 (ERK) [34] and calcium/calmodulin-dependent kinase II α (CaMKIα) [35] activity both of which are kinases known to regulate NKCC1 activity [33,36]. Both ERK [18] and CaMKIIα [37] are involved in referred hyperalgesia evoked by intracolonic capsaicin hence, an intriguing hypothesis is that IT NADA activates ERK and/or CaMKIIα leading to an increase in NKCC1 activity causing touch evoked pain. On the other hand, it is possible that spinal TRPV1 activation mediates allodynia by regulating GABAergic neurotransmission. In this regard, it has been shown that dorsal horn TRPV1 stimulation leads to a large increase in GABAergic network activity [38] which may be an alternative explanation for the role of NKCC1 in allodynia mediated by NADA. Clearly more work is needed to better understand the potential interplay between NKCC1 and TRPV1 and its role in allodynia; however, the anti-allodynic effects TRPV1 antagonists and NKCC1 inhibitors on the spinal level indicate that this might be a fruitful avenue for combined therapeutics.

## Conclusion

We have demonstrated that spinal NKCC1 blockade and spinal TRPV1 antagonism attenuates referred allodynia in response to a painful visceral stimulus. Moreover, we have shown that the endogenous TRPV1 agonist NADA, applied spinally, evokes stroking allodynia in the hindpaw that is TRPV1-dependent and inhibited by the NKCC1 blocker BUM. These findings further implicate NKCC1 in inflammatory allodynia and suggest that spinal TRPV1 receptors might be involved in regulating NKCC1 activity in nociceptive primary afferents.

## Methods

### Animals

Male C57BL6 mice between 20–25 g were used for all experiments. Experiments were in accordance with the Canadian Counsel on Animal Care (CCAC) and the International Association for the Study of Pain (IASP) guidelines for the care and use of experimental animals. All protocols were reviewed and approved by the McGill University Animal Care Committee.

### Behavioral Testing and Statistical Analysis

Mice were habituated to a Plexiglass testing box (10 × 10 × 8 cm) for 1–2 hours prior to testing. The model of referred allodynia has been described previously [19], briefly, just prior to application of CAP, petroleum jelly (Vaseline) was applied to the peri-anal area to avoid the stimulation of somatic areas by contact with the capsaicin. A total of 0.05 ml of 0.1% capsaicin solution was slowly injected via a fine cannula with a rounded tip (external diameter 0.61 mm), gently introduced 4 cm into the colon via the anus. Capsaicin (0.1%, Tocris, Ellisville, MO) was dissolved in 10% ethanol, 10% Tween 80 and 80% saline. Bumetanide (BUM; Sigma, St. Louis, MO), AMG 9810 (Tocris) and n-arachidonoyl- dopamine (NADA, Tocris) were dissolved in artificial cerebrospinal fluid (aCSF) vehicle. The aCSF vehicle was comprised of (in mM) 1.3 CaCl2 2H2O, 2.6 KCl, 0.9 MgCl, 21.0 NaHCO3, 2.5 Na2HPO47H2O, 125.0 NaCl, and 3.5 dextrose (pH 7.2–7.4). Intrathecal (IT) injections of BUM, AMG 9801 and NADA were made in 5 μl aCSF on lightly anesthetized mice by lumbar puncture at the L4–L5 level with a 30-gauge needle on a 50 μl Hamilton needle [39]. Referred visceral hyperalgesia was tested on the abdomen with calibrated von Frey filaments (1mN & 32 mN) at various time-points before or after intracolonic capsaicin treatment. Percent response frequency was calculated from the number of responses to 10 stimulations on the abdomen. Each filament was applied for approximately 1–2 s, with an inter-stimulus interval of 2–5 s. The appearance of any of the following behaviors on application of a filament to the abdomen was considered a withdrawal response: (i) sharp retraction of the abdomen, (ii) immediate licking or scratching of site of application of filament, or (iii) jumping. For experiments with NADA, mice were habituated as above and tested on the hindpaw with von Frey filaments of 1, 4, 8 and 16 mN using the same protocol as for abdominal testing. Mice were also tested for stroking allodynia by gently brushing the plantar surface of the hindpaw 10 times with a 5 sec inter-stimulus interval with a cotton bud (Q-tip). The following reactions were considered a withdrawal response to the cotton bud: (i) sharp retraction of hindpaw, (ii) immediate licking or scratching of site of application of the cotton bud, or (iii) jumping. Mice were used for only one experimental procedure and were humanely killed by anesthesia overdose immediately after testing. All testing was performed blind to condition and treatment. Data are shown as mean ± SEM and all analyses involved one or two way anova (indicated in figure captions) with bonferroni post-tests, where p < 0.05 was considered significant.

## Abbreviations

BUM, bumetanide; CaMKIIα, calcium/calmodulin-dependent kinase II α; DRR, dorsal root reflex; ERK, extracellular regulated kinase; KCC2, K^+^, Cl^- ^co-transporter; NADA, narachidonoyl- dopamine; NKCC1, Na^+^, K^+^, 2Cl^- ^co-transporter type 1; PAD, primary afferent depolarization; TRPV1, transient receptor potential vanilloid type 1.

## Competing interests

The author(s) declare that they have no competing interests.

## Authors' contributions

FC and TJP conceived the experiments; FC, MHP and TJP designed experiments; MHP, TJP and JME performed the experiments; TJP, MHP and FC wrote the manuscript. All authors read and approved the final manuscript.
